# Joint Association of Nicotinic Acetylcholine Receptor Variants with Abdominal Obesity in American Indians: The Strong Heart Family Study

**DOI:** 10.1371/journal.pone.0102220

**Published:** 2014-07-18

**Authors:** Yun Zhu, Jingyun Yang, Fawn Yeh, Shelley A. Cole, Karin Haack, Elisa T. Lee, Barbara V. Howard, Jinying Zhao

**Affiliations:** 1 Tulane University School of Public Health and Tropical Medicine, New Orleans, Louisiana, United States of America; 2 Rush Alzheimer's Disease Center, Rush University Medical Center, Chicago, Illinois, United States of America; 3 Center for American Indian Health Research, University of Oklahoma Health Science Center, Oklahoma City, Oklahoma, United States of America; 4 Texas Biomedical Research Institute, San Antonio, Texas, United States of America; 5 MedStar Health Research Institute, Hyattsville, Maryland, United States of America; 6 Georgetown and Howard Universities Centers for Translational Sciences, Washington, District of Columbia, United States of America; University of Michigan, United States of America

## Abstract

Cigarette smoke is a strong risk factor for obesity and cardiovascular disease. The effect of genetic variants involved in nicotine metabolism on obesity or body composition has not been well studied. Though many genetic variants have previously been associated with adiposity or body fat distribution, a single variant usually confers a minimal individual risk. The goal of this study is to evaluate the joint association of multiple variants involved in cigarette smoke or nicotine dependence with obesity-related phenotypes in American Indians. To achieve this goal, we genotyped 61 tagSNPs in seven genes encoding nicotine acetylcholine receptors (nAChRs) in 3,665 American Indians participating in the Strong Heart Family Study. Single SNP association with obesity-related traits was tested using family-based association, adjusting for traditional risk factors including smoking. Joint association of all SNPs in the seven nAChRs genes were examined by gene-family analysis based on weighted truncated product method (TPM). Multiple testing was controlled by false discovery rate (FDR). Results demonstrate that multiple SNPs showed weak individual association with one or more measures of obesity, but none survived correction for multiple testing. However, gene-family analysis revealed significant associations with waist circumference (p = 0.0001) and waist-to-hip ratio (p = 0.0001), but not body mass index (p = 0.20) and percent body fat (p = 0.29), indicating that genetic variants are jointly associated with abdominal, but not general, obesity among American Indians. The observed combined genetic effect is independent of cigarette smoking *per se*. In conclusion, multiple variants in the nAChR gene family are jointly associated with abdominal obesity in American Indians, independent of general obesity and cigarette smoking *per se*.

## Introduction

The global epidemics of overweight and obesity, and the resulting impact on diabetes, cardiovascular disease (CVD), and certain types of cancer [1]–[6] pose a great burden on public health. Obesity has a strong genetic predisposition, with heritability estimates ranging from 65–80%.[7] Genome-wide association studies (GWAS) and candidate gene studies have identified many genetic loci influencing body weight or body fat distribution, most of them have important role in the central nervous system.[8]–[13] However, inter-individual variation in body weight or body fat distribution attributable to each single variant was marginal. Collectively, the loci identified so far explain only a small proportion of disease variability, suggesting that additional genes with important roles remained to be discovered. It is well-accepted that the etiology of obesity is multifactorial involving multiple genes in biologically related pathways. A single gene with small or weak individual effect may not cause disease individually; instead, they act jointly in the context of networks or pathways in leading to disease susceptibility.[14] Therefore, a gene-family or pathway-based approach combining information from multiple genetic variants may capture a large proportion of the causal variants, and thus should have higher power than studying single gene alone in dissecting the complex genetic architecture of obesity or its related traits.

Cigarette smoking is strongly associated with body weight, body fat distribution, obesity and insulin resistance.[15], [16] However, the biological mechanisms linking smoking to body composition and obesity are incompletely understood. Cross-sectional studies indicate that cigarette smokers have lower body mass index (BMI) or body weight than nonsmokers, but heavy smokers are more likely to be overweight or obese.[17]–[21] In addition, smokers tend to be centrally obese and suffer from a greater risk of adverse metabolic consequences than nonsmokers.[15], [16] The differential metabolic effect of cigarette smoking on body fat distribution may imply inherent biological difference between different measures of body fat distribution. American Indians have the highest prevalence of cigarette smoking of all U. S. ethnic groups [22] and also suffer from high rates of diabetes and cardiovascular disease. It is unclear whether and how cigarette smoking contributes to body fat distribution in American Indians. Elucidation of the biological pathways underlying the association between smoking and body fat deposition has the potential to provide novel strategies for the prevention and intervention of obesity and related metabolic disorders in this ethnically important but traditionally understudied population.

Nicotine is the major bioactive component in cigarette smoke. It acts as a potent ganglionic and neural stimulant by binding to nicotinic acetylcholine receptors (*nAChRs*), a superfamily of ligand-gated ion channels that are widely present within neuronal and non-neuronal cell types.[23] Polymorphisms in genes encoding *nAChRs* have been associated with nicotine dependence [24] and lung cancer.[25] However, little research has been done to examine the potential impact of *nAChRs* variants on interindividual variability in body fat distribution. Moreover, existing studies primarily focused on single SNP analysis, which may not capture the joint contribution of multiple genes to disease risk. The goal of this study is to determine the joint association of 61 tagging SNPs in seven candidate genes involved in cigarette smoking with body fat distribution in a well-characterized population of American Indians participating in the Strong Heart Family Study.

## Methods

### Study population

The Strong Heart Family Study (SHFS) is a family-based prospective study designed to identify genetic factors for CVD, diabetes and associated risk factors in American Indians. A total of 3,665 tribal members aged 14–93 years from 94 multiplex families residing in Arizona (AZ), North and South Dakota (DK) and Oklahoma (OK) were recruited and examined between 2001 and 2003. Detailed descriptions of the SHFS protocols for the collection of phenotype data have been described previously.[26] All participants received a personal interview to collect data on demographic characteristics, medical history and lifestyle risk factors including smoking, alcohol consumption, diet and physical activity. A physical examination was given to each participant, including anthropometric and blood pressure measurements and an examination of the heart and lungs. Laboratory methods were reported previously.[26], [27] All study participants provided written consent. The SHFS protocol was approved by the Institutional Review Boards from the Indian Health Service and the participating centers.

### Anthropometric measurements for obesity-related traits

Anthropometric measurements included body weight, height and waist circumference (WC) measured with participants wearing light clothing and without shoes. Body mass index (BMI) was defined as body weight in kilograms divided by the square of the height in meters (kg/m^2^). Waist circumference was measured at the level of the umbilicus while the participant was supine. Hip circumference was measured at the level of widest circumference over greater trochanters with the legs close together. Waist-to-hip ratio (WHR) was calculated as waist circumference divided by hip circumference. Percent body fat (%BF) was estimated with an RJL impedance meter (Model B14101; RJL Equipment Company, Detroit, MI) using an equation based on total body water (M Singer, RJL Equipment Company, personal communication, 1992).

### Measurements of risk factors

Cigarette smoking was assessed *via* questionnaire. Participants were classified as current smokers, former smokers and nonsmokers. Current smokers reported smoking 100 or more cigarettes in their lifetime and were currently smoking every day or some days. Former smokers are those who had smoked 100 or more cigarettes but were no longer smoking. Nonsmokers are those who smoked fewer than 100 cigarettes or never smoked in their life time. Pack-years were calculated by multiplying the number of packs of cigarettes smoked per day by the number of years the person has smoked. Participants were categorized into current drinkers, former drinkers and never drinkers based on their history of alcohol consumption. Physical activity was assessed by the mean number of steps per day calculated by averaging the total number of steps recorded each day during the 7-day period. Dietary information was collected using the Block Food Frequency Questionnaire. According to the 1997 American Diabetes Association criteria, [28] diabetes was defined as fasting plasma glucose ≥7.0 mmol/L) or receiving insulin or oral hyperglycemic treatment. Impaired fasting glucose (IFG) was defined as a fasting glucose of 6.1–6.9 mmol/L, and fasting glucose <6.1 mmol/L is defined as normal.

### TagSNPs selection and genotyping

A total of 61 tagSNPs in seven nAChRs genes (*CHRNA3*-*A6*, *CHRNB2*-*B4*) from the *nAChRs* gene family were selected and genotyped in all SHFS participants. These genes were consistently reported to be associated with cigarette smoking in previous studies. TagSNPs were selected by the computer program Haploview 4.2.[29] Other factors were also considered in selection of SNPs, including minor allele frequency, SNP location, and Illumina design score. Detailed selection criteria were reported elsewhere.[30] All genotyping was done at the Texas Biomedical Research Institute using the Illumina VeraCode technology (Illumina, Inc., San Diego, CA). The average genotyping call rates were 98% for the chosen SNPs, and sample success rate was 99.5%.

### Statistical analysis

#### Single SNP association analysis

We tested the association of each individual SNP with BMI, WC, WHR or %BF (*all as continuous variables*) using family-based association tests by the computer program FBAT.[31] The following covariates were included as covariates: age, sex, study center, smoking status (ever vs. never smoker), alcohol consumption (current vs. former vs. never), physical activity, total daily energy intake, socioeconomic status, and history of diabetes. Continuous variables were logarithmically transformed to improve normality. Participants with missing information on smoking status (n = 15) or covariates (n = 10) were excluded from statistical analyses, resulting in a final sample size of 3,640 in the statistical analysis. Multiple testing in the individual SNP association analysis and gene-based analysis was corrected using the Storey's q-value method.[32]

#### Gene-based and gene-family association analysis

The association between a candidate gene (including all SNPs within that gene) and body composition was assessed by combining p-values from single SNP association analysis. This was done by weighted truncated product method (wTPM)[33] using effect size of each SNP as weight. P values of wTPM were estimated by 50,000 Monte Carlo simulations, as described previously.[33] Gene-family analysis was performed by combining p-values of each candidate gene obtained from gene-based analysis including all seven genes in the nAChRs gene family. Detailed methods for gene-based and gene-family association analyses have been described previously.[30], [34]

#### Sensitivity analyses

To examine the impact of diabetes on the association between genetic variants and body fat distribution, we conducted additional analysis by excluding participants with diabetes status. To determine whether cigarette smoking influences the association between genetic variants and body composition, we compared results before and after adjustment for smoking status. In addition, we conducted analysis to examine whether the association of genetic variants with central obesity is independent of general obesity by additional adjusting for BMI. To investigate whether results from gene-based or gene-family analysis are primarily driven by the most significant SNPs, we conducted additional analysis by removing the most significant SNP from statistical analyses. All analyses were done using *R* 2.15.2 and SAS 9.2 (SAS Institute Inc., Cary, NC).

## Results

### Baseline characteristics of the study participants

**Table 1** presents the baseline characteristics of the study participants according to smoking status. Compared to never smokers, ever smokers (current plus former smoker) were older, more likely to be males, and had higher levels of total cholesterol and triglyceride. In addition, ever smokers had significantly larger WHR than never smokers, suggesting that they are more likely to be centrally obese. No significant difference in BMI, WC or %BF was observed between ever smokers and never smokers.

**Table 1:** Characteristics of study participants according to smoking status (n = 3,640).

	Ever smoker (n = 2116) (mean ± SD or %)	Never smoker (n = 1524) (mean ± SD or %))	P^‡^
Age (years)			
Mean	41.2±15.6	37.9±18.5	<0.0001
Range	14.09–93.25	15.45–90.84	
Male sex (%)	44.5	33.9	<0.0001
Type 2 Diabetes (%)	24.2	21.0	0.49
Body mass index (kg/m^2^)	32.2±7.9	32.3±7.9	0.72
Waist circumference (cm)	105.3±18.2	103.6±19.1	0.20
Waist-to-hip ratio	0.92±0.08	0.90±0.08	0.002
Percent body fat	36.8±10.0	38.0±10.4	0.62
Systolic blood pressure (mmHg)	123.4±17.0	121.7±17.3	0.39
Diastolic blood pressure(mmHg)	76.8±10.9	75.5±11.5	0.17
High-density lipoprotein (mg/dL)	50.5±14.5	51.3±14.7	0.44
Low-density lipoprotein (mg/dL)	99.4±29.7	96.4±28.9	0.30
Plasma hsCRP (mg/L)	7.0±9.2	6.8±9.9	0.45
eGFR (ml/min/1.73 m^2^)	99.1±27.2	101.8±30.1	0.96
Total cholesterol (mg/dL)	183.4±38.6	177.2±34.8	0.006
Total triglyceride (mg/dL)	177.2±197.3	155.4±123.8	0.01
Fasting glucose (mg/dL)	115.8±52.7	112.1±52.8	0.52
Insulin (uU/mL)	18.7±20.2	18.8±20.4	0.74

^*^P values were obtained by GEE, adjusting for age and sex when appropriate; ^‡^ Former plus current smokers.

### Association of individual SNP with body composition

The pattern of linkage disequilibrium (LD) and allele frequencies of the studied SNPs stratified by study center (AZ, OK and DK) were described previously.[30] Results for single SNP association were listed in **Table 2**, which shows that multiple SNPs were marginally associated with one or more body composition measures, but none survived correction for multiple testing.

**Table 2:** P-values of single SNP association analysis for the 61 SNPs with obesity measures by FBAT (n = 3,640).

SNP	Gene	BMI	WC	WHR	%BF	SNP	Gene	BMI	WC	WHR	%BF
rs1051730	*CHRNA3*	0.1877	0.0412	0.0365	0.8535	rs905739	*CHRNA5*	0.3011	0.1160	0.3512	0.4066
rs11637630	*CHRNA3*	0.2825	0.2414	0.3157	0.5119	rs951266	*CHRNA5*	0.3051	0.0853	0.1057	0.5417
rs12910984	*CHRNA3*	0.2631	0.1942	0.2390	0.5500	rs2304297	*CHRNA6*	0.6362	0.4043	0.3585	0.3681
rs12914385	*CHRNA3*	0.0980	0.0218	0.0178	0.1244	rs2072658	*CHRNB2*	0.1954	0.1876	0.3787	0.6123
rs1317286	*CHRNA3*	0.0915	0.0167	0.0117	0.5951	rs2072659	*CHRNB2*	0.3214	0.1891	0.0823	0.2303
rs1878399	*CHRNA3*	0.4281	0.8369	0.6572	0.3977	rs2072660	*CHRNB2*	0.8459	0.5275	0.0926	0.2502
rs3743074	*CHRNA3*	0.3945	0.3921	0.2356	0.1417	rs2072661	*CHRNB2*	0.7452	0.4177	0.0584	0.2765
rs3743078	*CHRNA3*	0.3160	0.2671	0.3977	0.6535	rs3811450	*CHRNB2*	0.1970	0.1414	0.0260	0.1095
rs578776	*CHRNA3*	0.3284	0.2962	0.5690	0.1495	rs10958726	*CHRNB3*	0.0594	0.0225	0.1464	0.0168
rs6495308	*CHRNA3*	0.3171	0.2105	0.1912	0.5500	rs13277254	*CHRNB3*	0.1179	0.0535	0.2173	0.0491
rs660652	*CHRNA3*	0.6294	0.6067	0.3738	0.2455	rs13280604	*CHRNB3*	0.1150	0.0479	0.2607	0.0513
rs7177514	*CHRNA3*	0.2710	0.1923	0.2423	0.5869	rs4950	*CHRNB3*	0.1437	0.0542	0.2759	0.0635
rs2236196	*CHRNA4*	0.2525	0.1869	0.1944	0.6613	rs4952	*CHRNB3*	0.0470	0.1484	0.5240	0.1034
rs2273504	*CHRNA4*	0.1865	0.2433	0.5767	0.1302	rs4953	*CHRNB3*	0.0470	0.1484	0.5240	0.1034
rs3787116	*CHRNA4*	0.1821	0.2634	0.2975	0.1245	rs4954	*CHRNB3*	0.6603	0.5589	0.9305	0.3235
rs3787137	*CHRNA4*	0.4825	0.4961	0.1098	0.1499	rs6474413	*CHRNB3*	0.0741	0.0284	0.1514	0.0257
rs6122429	*CHRNA4*	0.1202	0.2037	0.8469	0.2186	rs11633223	*CHRNB4*	0.1690	0.2089	0.2744	0.5478
rs11633585	*CHRNA5*	0.3977	0.4575	0.5277	0.9635	rs11636605	*CHRNB4*	0.1269	0.1615	0.1820	0.2898
rs11637635	*CHRNA5*	0.6294	0.5317	0.3256	0.2455	rs12440014	*CHRNB4*	0.9045	0.7610	0.3659	0.6559
rs16969968	*CHRNA5*	0.1503	0.0381	0.0344	0.7120	rs12914008	*CHRNB4*	0.7220	0.5278	0.1373	0.6542
rs17483686	*CHRNA5*	0.0221	0.0257	0.2607	0.2522	rs1316971	*CHRNB4*	0.1115	0.1543	0.1947	0.2898
rs17486278	*CHRNA5*	0.1903	0.0605	0.0660	0.7120	rs16970006	*CHRNB4*	0.3634	0.2398	0.2761	0.2166
rs2036527	*CHRNA5*	0.1006	0.0228	0.0161	0.6179	rs17487223	*CHRNB4*	0.3746	0.3187	0.1100	0.0967
rs514743	*CHRNA5*	0.6566	0.5117	0.3174	0.2507	rs1948	*CHRNB4*	0.3430	0.7518	0.4674	0.5464
rs569207	*CHRNA5*	0.3125	0.2745	0.3082	0.7889	rs1996371	*CHRNB4*	0.0797	0.0216	0.0118	0.3807
rs588765	*CHRNA5*	0.7221	0.8917	0.5775	0.3476	rs3813567	*CHRNB4*	0.1407	0.0573	0.0996	0.5025
rs615470	*CHRNA5*	0.4599	0.6513	0.5450	0.1476	rs3971872	*CHRNB4*	0.1438	0.3257	0.2571	0.7550
rs637137	*CHRNA5*	0.3166	0.2928	0.3302	0.8278	rs7178270	*CHRNB4*	0.4334	0.3123	0.1807	0.5865
rs680244	*CHRNA5*	0.6953	0.6374	0.4410	0.5823	rs8023462	*CHRNB4*	0.4391	0.8614	0.2181	0.4958
rs684513	*CHRNA5*	0.9469	0.7493	0.6940	0.7512	rs950776	*CHRNB4*	0.6998	0.4449	0.0702	0.8830
rs8034191	*CHRNA5*	0.1258	0.0199	0.0187	0.3158						

All P-values adjusted for age, sex, study center, smoking status, alcohol intake, levels of physical activity, total daily energy intake, social economics status and diabetes status.

### Gene-based and gene-family association with obesity-related traits

**Table 3** presents the results for gene-based and gene-family association analyses. For gene-based analysis, genetic variants in *CHRNA5* were significantly associated with waist circumference and marginally associated with WHR. Another three genes, *CHRNA3, CHRNB3* and *CHRNB4*, showed weak association with waist circumference and/or WHR. The gene-family comprising all seven nAChRs genes showed significant associations with both waist circumference and WHR. However, no association, either gene-based or gene-family analysis, was observed for BMI or percent body fat.

**Table 3:** Gene-based and gene-family association of nAChRs variants with obesity (n = 3,640).

	BMI	WC	WHR	%BF
CHRNA3	0.1730	0.0360	0.0320	0.4870
CHRNA4	0.2380	0.2220	0.2150	0.1460
CHRNA5	0.3050	**0.0001**	0.0160	0.5870
CHRNA6	0.6352	0.4033	0.3575	0.3671
CHRNB2	0.3840	0.3010	0.0190	0.2110
CHRNB3	0.0470	0.0370	0.1980	0.0300
CHRNB4	0.1890	0.0560	0.0950	0.3310
Gene-family association	0.1970	**0.0001**	**0.0001**	0.2910

P-values in bold indicates significant association after adjusting for multiple testing by FDR.

### Results for sensitivity analysis

Given the strong correlation between obesity and diabetes, we examined the potential impact of diabetes on the association of the nAChR gene family with obesity measures by excluding participants with diabetes (**Tables S1**). It shows that the nAChRs gene family remained to be significantly associated with abdominal (central) obesity among normoglycemic subjects, suggesting that the observed genetic association was not driven by diabetes. Similar results for gene-family analysis were observed before and after adjustments for smoking status or pack-years, suggesting that smoking *per se* did not affect the observed genetic associations with abdominal fat distribution. Moreover, this association was independent of general obesity because additionally adjustment for BMI did not attenuate the gene-family association (**Table S2**). In addition, removing the most significant SNPs from gene-family analysis did not change our results, indicating that the observed gene-family association may not be driven by SNPs showing the most significant association with WC or WHR (**Table S3**).

## Discussion

In this study, we demonstrated for the first time that multiple SNPs in the nAChRs gene family, each of which has a small individual effect, exhibited joint association with abdominal obesity among American Indians participating in the SHFS. This association is independent of body mass index and cigarette smoking *per se.* Although the potential role of this pathway in abdominal obesity awaits further research, the identified genetic pathway appears to highlight novel biological mechanisms involved in the regulation of central adiposity in American Indians.

It is generally accepted that BMI and percent body fat are measures of the overall level of adiposity or general obesity, whereas waist circumference and WHR estimate the amount of visceral or abdominal fat, and are indicators of central obesity.[35] Our finding for an association of the nAChRs gene family with WC and WHR, but not BMI and percent body fat, is in agreement with previous studies demonstrating an association of smoking with abdominal or visceral obesity but not overall fatness,[16], [36], [37] and suggested a differential effect of the nAChRs pathway on body fat distribution among American Indians. Previous studies have reported a differential negative effect of general or central fat accumulation on cardiometabolic risk, with abdominal or visceral fat showing particularly adverse consequences on health.[38] This suggests that different measures of body fat composition may have different pathophysiological roles in leading to cardiometabolic risk. Moreover, genome-wide association studies (GWAS) have identified multiple genetic loci associated with body fat distribution, but there was incomplete overlap with the loci influencing central obesity (e.g.,WC, WHR) and general obesity (e.g., BMI, %BF), implying that physiological variation in abdominal fat accumulation and overall level of adiposity may attribute to different susceptibility variants. The observed differential effect of the nAChRs genetic pathway on central obesity but not general obesity in our study lends further support for a potential different genetic impact of the nAChRs variants on body fat distribution in American Indians. In a previous study, we observed that nAChRs genetic pathway is associated with type 2 diabetes.[30] Moreover, among those diagnosed with type 2 diabetes, more than 93% were also overweight. These findings suggest that obesity and diabetes may share some same genetic background, consistent with previous studies[39], [40].

The link between cigarette smoking and body weight is evident, but the mechanisms through which smoking influences body weight or body fat distribution are complex and incompletely understood. Body weight is determined by daily calorie intake and energy expenditure. Nicotine in cigarette smoke may influence body weight by decreasing appetite, and hence calorie intake, or by raising metabolic rate or decreasing metabolic efficiency through its potential effects on central nervous system.[41] For example, a recent study shows that smoking stimulates the activity of pro-opiomelanocortin via activation of hypothalamic α_3_β_4_ nAChRs.[42] This process may also involve the stimulation of the sympathetic nervous system,[43] leading to the release of neurotransmitters such as higher cortisol concentrations,[44] thereby influencing visceral or abdominal adiposity.[45] These results demonstrated neural control of cigarette smoking on body fat distribution or related phenotypes. In line with these findings, the association of the nicotine genetic pathway with abdominal adiposity identified in our study is independent of cigarette smoking *per se*, lending further support for a role of the central nerve system in mediating the association between cigarette smoking and body fat distribution.

Several aspects of our study merit comments. First, consistent with previous research,[46] our results demonstrated that a single SNP may show no or minimal individual association with a complex trait, but the joint effect of multiple SNPs within a gene or a biological pathway on disease susceptibility could be large. For example, after correction for multiple testing, no variant in *CHRNA5* was individually associated with any of the examined obesity measures, but gene-based analysis showed that this gene was significantly associated with waist circumference. The pathway as a whole was also significantly associated with both WC and WHR. This reinforces the emerging evidence that it is the cumulative effect of many loci that underlies susceptibility to disease. In addition, our results emphasize the important role of statistical approaches that captures the joint contribution of multiple variants simultaneously in better characterizing the complex genetic etiology of common diseases. Second, the gene cluster *CHRNA3/CHRNA5/CHRNB4*, located on chromosome 15q24, has previously been associated with smoking quantity and nicotine dependence.[47], [48] In our study, the *CHRNA5* gene was significantly associated with WC, but *CHRNA3* and *CHRNB4* showed no or weak association with WC or WHR, and none of the three genes was associated with BMI. In a recent meta-analysis, rs1051730 in *CHRNA3* was significantly associated with BMI among ever smokers but not never smokers, indicating a gene × smoking interaction on BMI.[49] However, we did not observe any effect of this SNP on BMI. These discrepancies are probably attributable to the different genetic background between American Indians and other ethnic populations. Alternatively, these variants may influence obesity susceptibility through pathways beyond cigarette smoking in our sample. Third, the observed pathway association with abdominal fat distribution is unlikely to be mediated by cigarettes smoking *per se* because all our statistical analyses controlled for smoking status. Finally, we observed a joint genetic association with central but not peripheral obesity, and this association is independent of overall adiposity. These findings provide initial evidence for a differential effect of the nAChRs variants on body fat distribution, and may also shed light on the pathophysiological mechanisms underlying the association between cigarette smoking, body fat distribution and cardiometabolic risk.

There are limitations with the study. First, although we have controlled many potential confounders, we cannot rule out the possibility of residual confounding by other unknown or unmeasured factors. Second, based on LD analyses, it appears that subjects from different study center have heterogeneous genetic background, suggesting a possible population admixture among our study participants. However, this should not be an issue for our analysis because we used family-based association analyses, which are robust to population substructure.[31] Third, our study is cross-sectional which precludes any causal inference. Fourth, previous studies indicated that heavy smokers tend to have higher body weight than light or nonsmokers. Our analyses, however, did not differentiate the heaviness of smoking due to lack of detailed clinical information on smoking quantity. In addition, our analysis focused on common genetic variants, but low frequency and/or rare variants may have large effects on disease susceptibility and thus should be investigated in future research. Finally, our analyses were undertaken among a cohort of American Indians with high prevalence of cigarette smoking and diabetes, and with different genetic architecture from other ethnic groups. It is unclear whether our results can be generalized to other ethnic groups with different patterns of body fat distribution or risk profiles.

In summary, this study provides initial evidence that multiple genetic variants in the nAChRs gene family jointly are jointly associated with central body fat distribution in American Indians. The impact of these genetic variants on the susceptibility to abdominal obesity may not be mediated by cigarette smoking *per se*. Our results may provide novel insights into obesity etiology and also provides valuable information for personalized obesity prevention or intervention in American Indians who suffer from increasingly high prevalence of obesity and diabetes.

## Supporting Information

Table S1 **Gene-based and gene-family associations of nAChRs variants with obesity among subjects without diabetes by wTPM (n = 2,830).**
(DOCX)

Table S2 **Gene-based and gene-family associations of the nAChRs variants with central obesity after additional adjustment for BMI (n = 3,640).**
(DOCX)

Table S3 Gene-based and gene-family associations of nAChRs variants with obesity measures by wTPM after removing most significant SNPs^*^ (n = 3,640).
(DOCX)

## References

[1] Hubert HB, Feinleib M, McNamara PM, Castelli WP (1983) Obesity as an independent risk factor for cardiovascular disease: a 26-year follow-up of participants in the Framingham Heart Study. Circulation 67: 968–977. 6219830 10.1161/01.cir.67.5.968

[2] Goran MI, Ball GD, Cruz ML (2003) Obesity and risk of type 2 diabetes and cardiovascular disease in children and adolescents. J Clin Endocrinol Metab 88: 1417–1427. 12679416 10.1210/jc.2002-021442

[3] Chan JM, Rimm EB, Colditz GA, Stampfer MJ, Willett WC (1994) Obesity, fat distribution, and weight gain as risk factors for clinical diabetes in men. Diabetes Care 17: 961–969. 7988316 10.2337/diacare.17.9.961

[4] Sorof J, Daniels S (2002) Obesity hypertension in children: a problem of epidemic proportions. Hypertension 40: 441–447. 12364344 10.1161/01.hyp.0000032940.33466.12

[5] Rahmouni K, Correia ML, Haynes WG, Mark AL (2005) Obesity-associated hypertension: new insights into mechanisms. Hypertension 45: 9–14. 15583075 10.1161/01.HYP.0000151325.83008.b4

[6] Calle EE, Thun MJ (2004) Obesity and cancer. Oncogene 23: 6365–6378. 15322511 10.1038/sj.onc.1207751

[7] Malis C, Rasmussen EL, Poulsen P, Petersen I, Christensen K, et al (2005) Total and regional fat distribution is strongly influenced by genetic factors in young and elderly twins. Obes Res 13: 2139–2145. 16421348 10.1038/oby.2005.265

[8] Speliotes EK, Willer CJ, Berndt SI, Monda KL, Thorleifsson G, et al (2010) Association analyses of 249,796 individuals reveal 18 new loci associated with body mass index. Nat Genet 42: 937–948. 20935630 10.1038/ng.686 PMC3014648

[9] Lindgren CM, Heid IM, Randall JC, Lamina C, Steinthorsdottir V, et al (2009) Genome-wide association scan meta-analysis identifies three Loci influencing adiposity and fat distribution. PLoS Genet 5: e1000508. 19557161 10.1371/journal.pgen.1000508 PMC2695778

[10] Heid IM, Jackson AU, Randall JC, Winkler TW, Qi L, et al (2010) Meta-analysis identifies 13 new loci associated with waist-hip ratio and reveals sexual dimorphism in the genetic basis of fat distribution. Nat Genet 42: 949–960. 20935629 10.1038/ng.685 PMC3000924

[11] Bradfield JP, Taal HR, Timpson NJ, Scherag A, Lecoeur C, et al (2012) A genome-wide association meta-analysis identifies new childhood obesity loci. Nat Genet 44: 526–531. 22484627 10.1038/ng.2247 PMC3370100

[12] Yeo GS, Connie Hung CC, Rochford J, Keogh J, Gray J, et al (2004) A de novo mutation affecting human TrkB associated with severe obesity and developmental delay. Nat Neurosci 7: 1187–1189. 15494731 10.1038/nn1336

[13] Gray J, Yeo GS, Cox JJ, Morton J, Adlam AL, et al (2006) Hyperphagia, severe obesity, impaired cognitive function, and hyperactivity associated with functional loss of one copy of the brain-derived neurotrophic factor (BDNF) gene. Diabetes 55: 3366–3371. 17130481 10.2337/db06-0550 PMC2413291

[14] Schadt EE (2009) Molecular networks as sensors and drivers of common human diseases. Nature 461: 218–223. 19741703 10.1038/nature08454

[15] Chiolero A, Faeh D, Paccaud F, Cornuz J (2008) Consequences of smoking for body weight, body fat distribution, and insulin resistance. Am J Clin Nutr 87: 801–809. 18400700 10.1093/ajcn/87.4.801

[16] Clair C, Chiolero A, Faeh D, Cornuz J, Marques-Vidal P, et al (2011) Dose-dependent positive association between cigarette smoking, abdominal obesity and body fat: cross-sectional data from a population-based survey. BMC Public Health 11: 23. 21223575 10.1186/1471-2458-11-23 PMC3025841

[17] Shimokata H, Muller DC, Andres R (1989) Studies in the Distribution of Body Fat. III. Effects of cigarette smoking. JAMA 261: 1169–1173. 2915440

[18] Flegal KM, Troiano RP, Pamuk ER, Kuczmarski RJ, Campbell SM (1995) The influence of smoking cessation on the prevalence of overweight in the United States. N Engl J Med 333: 1165–1170. 7565970 10.1056/NEJM199511023331801

[19] Bamia C, Trichopoulou A, Lenas D, Trichopoulos D (2004) Tobacco smoking in relation to body fat mass and distribution in a general population sample. Int J Obes Relat Metab Disord 28: 1091–1096. 15197410 10.1038/sj.ijo.0802697

[20] John U, Hanke M, Rumpf HJ, Thyrian JR (2005) Smoking status, cigarettes per day, and their relationship to overweight and obesity among former and current smokers in a national adult general population sample. Int J Obes (Lond) 29: 1289–1294. 15997244 10.1038/sj.ijo.0803028

[21] Chiolero A, Jacot-Sadowski I, Faeh D, Paccaud F, Cornuz J (2007) Association of cigarettes smoked daily with obesity in a general adult population. Obesity (Silver Spring) 15: 1311–1318. 17495208 10.1038/oby.2007.153

[22] Daley CM, Greiner KA, Nazir N, Daley SM, Solomon CL, et al (2010) All Nations Breath of Life: using community-based participatory research to address health disparities in cigarette smoking among American Indians. Ethn Dis 20: 334–338. 21305818 PMC3061617

[23] Millar NS, Harkness PC (2008) Assembly and trafficking of nicotinic acetylcholine receptors (Review). Mol Membr Biol 25: 279–292. 18446614 10.1080/09687680802035675

[24] Changeux JP (2010) Nicotine addiction and nicotinic receptors: lessons from genetically modified mice. Nat Rev Neurosci 11: 389–401. 20485364 10.1038/nrn2849

[25] Saccone NL, Culverhouse RC, Schwantes-An TH, Cannon DS, Chen X, et al (2010) Multiple independent loci at chromosome 15q25.1 affect smoking quantity: a meta-analysis and comparison with lung cancer and COPD. PLoS Genet 6 e1001053. 10.1371/journal.pgen.1001053 PMC2916847 20700436

[26] Lee ET, Welty TK, Fabsitz R, Cowan LD, Le NA, et al (1990) The Strong Heart Study. A study of cardiovascular disease in American Indians: design and methods. Am J Epidemiol 132: 1141–1155. 2260546 10.1093/oxfordjournals.aje.a115757

[27] Howard BV, Welty TK, Fabsitz RR, Cowan LD, Oopik AJ, et al (1992) Risk factors for coronary heart disease in diabetic and nondiabetic Native Americans. The Strong Heart Study. Diabetes 41 Suppl 2: 4–11. 1526334 10.2337/diab.41.2.s4

[28] Expert Committee on the Diagnosis and Classification of Diabetes Mellitus (2003) Report of the Expert Committee on the Diagnosis and Classification of Diabetes Mellitus. Diabetes Care Suppl 1: s5–s20. 12502614 10.2337/diacare.26.2007.s5

[29] Barrett JC, Fry B, Maller J, Daly MJ (2005) Haploview: analysis and visualization of LD and haplotype maps. Bioinformatics 21: 263–265. 15297300 10.1093/bioinformatics/bth457

[30] Yang J, Zhu Y, Cole SA, Haack K, Zhang Y, et al (2012) A gene-family analysis of 61 genetic variants in the nicotinic acetylcholine receptor genes for insulin resistance and type 2 diabetes in American Indians. Diabetes 61: 1888–1894. 22586585 10.2337/db11-1393 PMC3379651

[31] Horvath S, Xu X, Laird NM (2001) The family based association test method: strategies for studying general genotype—phenotype associations. Eur J Hum Genet 9: 301–306. 11313775 10.1038/sj.ejhg.5200625

[32] Storey JD, Tibshirani R (2003) Statistical significance for genomewide studies. Proc Natl Acad Sci U S A 100: 9440–9445. 12883005 10.1073/pnas.1530509100 PMC170937

[33] Sheng X, Yang J (2013) Truncated Product Methods for Panel Unit Root Tests. Oxf Bull Econ Stat 75: 624–636. 23869116 10.1111/j.1468-0084.2012.00705.x PMC3711747

[34] Yang J, Zhu Y, Lee ET, Zhang Y, Cole SA, et al (2013) Joint associations of 61 genetic variants in the nicotinic acetylcholine receptor genes with subclinical atherosclerosis in American Indians: a gene-family analysis. Circ Cardiovasc Genet 6: 89–96. 23264444 10.1161/CIRCGENETICS.112.963967 PMC3878644

[35] Flegal KM, Shepherd JA, Looker AC, Graubard BI, Borrud LG, et al (2009) Comparisons of percentage body fat, body mass index, waist circumference, and waist-stature ratio in adults. Am J Clin Nutr 89: 500–508. 19116329 10.3945/ajcn.2008.26847 PMC2647766

[36] Kim JH, Shim KW, Yoon YS, Lee SY, Kim SS, et al (2012) Cigarette smoking increases abdominal and visceral obesity but not overall fatness: an observational study. PLoS One 7: e45815. 23029258 10.1371/journal.pone.0045815 PMC3454366

[37] Yun JE, Kimm H, Choi YJ, Jee SH, Huh KB (2012) Smoking is associated with abdominal obesity, not overall obesity, in men with type 2 diabetes. J Prev Med Public Health 45: 316–322. 23091657 10.3961/jpmph.2012.45.5.316 PMC3469814

[38] Snijder MB, van Dam RM, Visser M, Seidell JC (2006) What aspects of body fat are particularly hazardous and how do we measure them? Int J Epidemiol 35: 83–92. 16339600 10.1093/ije/dyi253

[39] Ng MC, Park KS, Oh B, Tam CH, Cho YM, et al (2008) Implication of genetic variants near TCF7L2, SLC30A8, HHEX, CDKAL1, CDKN2A/B, IGF2BP2, and FTO in type 2 diabetes and obesity in 6,719 Asians. Diabetes 57: 2226–2233. 18469204 10.2337/db07-1583 PMC2494677

[40] McCarthy MI (2010) Genomics, type 2 diabetes, and obesity. N Engl J Med 363: 2339–2350. 21142536 10.1056/NEJMra0906948

[41] Audrain-McGovern J, Benowitz NL (2011) Cigarette smoking, nicotine, and body weight. Clin Pharmacol Ther 90: 164–168. 21633341 10.1038/clpt.2011.105 PMC3195407

[42] Mineur YS, Abizaid A, Rao Y, Salas R, DiLeone RJ, et al (2011) Nicotine decreases food intake through activation of POMC neurons. Science 332: 1330–1332. 21659607 10.1126/science.1201889 PMC3113664

[43] Hofstetter A, Schutz Y, Jequier E, Wahren J (1986) Increased 24-hour energy expenditure in cigarette smokers. N Engl J Med 314: 79–82. 3941694 10.1056/NEJM198601093140204

[44] Yoshida T, Sakane N, Umekawa T, Kogure A, Kondo M, et al (1999) Nicotine induces uncoupling protein 1 in white adipose tissue of obese mice. Int J Obes Relat Metab Disord 23: 570–575. 10411229 10.1038/sj.ijo.0800870

[45] Pasquali R, Vicennati V (2000) Activity of the hypothalamic-pituitary-adrenal axis in different obesity phenotypes. Int J Obes Relat Metab Disord 24 Suppl 2: S47–49. 10997608

[46] Low YL, Li Y, Humphreys K, Thalamuthu A, Darabi H, et al (2010) Multi-variant pathway association analysis reveals the importance of genetic determinants of estrogen metabolism in breast and endometrial cancer susceptibility. PLoS Genet 6: e1001012. 20617168 10.1371/journal.pgen.1001012 PMC2895650

[47] Saccone NL, Wang JC, Breslau N, Johnson EO, Hatsukami D, et al (2009) The CHRNA5-CHRNA3-CHRNB4 nicotinic receptor subunit gene cluster affects risk for nicotine dependence in African-Americans and in European-Americans. Cancer Res 69: 6848–6856. 19706762 10.1158/0008-5472.CAN-09-0786 PMC2874321

[48] Li MD, Xu Q, Lou XY, Payne TJ, Niu T, et al (2010) Association and interaction analysis of variants in CHRNA5/CHRNA3/CHRNB4 gene cluster with nicotine dependence in African and European Americans. Am J Med Genet B Neuropsychiatr Genet 153B: 745–756. 19859904 10.1002/ajmg.b.31043 PMC2924635

[49] Freathy RM, Kazeem GR, Morris RW, Johnson PC, Paternoster L, et al (2011) Genetic variation at CHRNA5-CHRNA3-CHRNB4 interacts with smoking status to influence body mass index. Int J Epidemiol 40: 1617–1628. 21593077 10.1093/ije/dyr077 PMC3235017

