# Supplementary material for: Joint Association of Nicotinic Acetylcholine Receptor Variants with Abdominal Obesity in American Indians: The Strong Heart Family Study
Source: PLoS One. 2014 Jul 18;9(7):e102220. doi: 10.1371/journal.pone.0102220 (PMC4103845; doi:10.1371/journal.pone.0102220)
Supplement: Table S1 — Gene-based and gene-family associations of nAChRs variants with obesity among subjects without diabetes by wTPM (n = 2,830). (DOCX) [file pone.0102220.s001.docx]

**Table S1.** Gene-based and gene-family associations of nAChRs variants with obesity among subjects without diabetes by wTPM (n=2,830)

|  | **BMI** | | **WC** | **WHR** | | **%BF** |
| --- | --- | --- | --- | --- | --- | --- |
| *CHRNA3* | 0.1900 | | 0.0680 | 0.0710 | 0.3900 | |
| *CHRNA4* | 0.4920 | | 0.3960 | 0.1160 | 0.2690 | |
| *CHRNA5* | 0.1510 | | 0.0200 | 0.0240 | 0.4550 | |
| *CHRNA6* | 0.4824 | | 0.3201 | 0.6758 | 0.2629 | |
| *CHRNB2* | 0.2300 | | 0.1750 | 0.1070 | 0.1810 | |
| *CHRNB3* | 0.1580 | | 0.0340 | 0.0150 | 0.0270 | |
| *CHRNB4* | 0.0650 | | 0.0180 | 0.0630 | 0.3830 | |
| Gene-family association | | 0.1980 | **0.0003** | **0.0046** | 0.2780 | |
| P-values in bold indicates significant association after adjusting for multiple testing by FDR | | | | | | |
