# Supplementary material for: Joint Association of Nicotinic Acetylcholine Receptor Variants with Abdominal Obesity in American Indians: The Strong Heart Family Study
Source: PLoS One. 2014 Jul 18;9(7):e102220. doi: 10.1371/journal.pone.0102220 (PMC4103845; doi:10.1371/journal.pone.0102220)
Supplement: Table S2 — Gene-based and gene-family associations of the nAChRs variants with central obesity after additional adjustment for BMI (n = 3,640). (DOCX) [file pone.0102220.s002.docx]

**Table S2.** Gene-based and gene-family associations of the nAChRs variants with central obesity after additional adjustment for BMI (n=3,640)

|  | **WC** | **WHR** |  |
| --- | --- | --- | --- |
| *CHRNA3* | 0.0377 | 0.0337 |  |
| *CHRNA4* | 0.1622 | 0.2923 |  |
| *CHRNA5* | **0.0002** | 0.0146 |  |
| *CHRNA6* | 0.2981 | 0.4234 |  |
| *CHRNB2* | 0.3641 | 0.0215 |  |
| *CHRNB3* | 0.0534 | 0.2086 |  |
| *CHRNB4* | 0.0649 | 0.0593 |  |
| Gene-family association | **0.0001** | **0.0001** |  |
| P-values in bold indicates significant association after adjusting for multiple testing by FDR. | | | |
