# Supplementary material for: Joint Association of Nicotinic Acetylcholine Receptor Variants with Abdominal Obesity in American Indians: The Strong Heart Family Study
Source: PLoS One. 2014 Jul 18;9(7):e102220. doi: 10.1371/journal.pone.0102220 (PMC4103845; doi:10.1371/journal.pone.0102220)
Supplement: Table S3 — Gene-based and gene-family associations of nAChRs variants with obesity measures by wTPM after removing most significant SNPs* (n = 3,640). (DOCX) [file pone.0102220.s003.docx]

**Table S3.** Gene-based and gene-family associations of nAChRs variants with obesity measures by wTPM after removing most significant SNPs^*^ (n=3,640)

|  | **BMI** | | **WC** | **WHR** | **%BF** |
| --- | --- | --- | --- | --- | --- |
| *CHRNA3* | | 0.2120 | 0.1240 | 0.0960 | 0.4460 |
| *CHRNA4* | | 0.2420 | 0.2480 | 0.2030 | 0.1500 |
| *CHRNA5* | | 0.2870 | **0.0002** | 0.0650 | 0.5690 |
| *CHRNA6* | | 0.6352 | 0.4033 | 0.3575 | 0.3671 |
| *CHRNB2* | | 0.3710 | 0.2970 | 0.0230 | 0.2250 |
| *CHRNB3* | | 0.0570 | 0.0420 | 0.3290 | 0.0500 |
| *CHRNB4* | | 0.4310 | 0.3400 | 0.1700 | 0.3450 |
| Gene-family association | | 0.2340 | **0.0070** | **0.0120** | 0.3920 |
| ^*^rs1317286 in *CHRNA3*, rs16969968 in *CHRNA5*, rs10958726 in *CHRNB3* and rs1996371 in *CHRNB4*.  P-values in bold indicates significant association after adjusting for multiple testing by FDR. | | | | | |
